# Multicystic urothelial carcinoma of the bladder with gland-like lumina and with signet-ring cells. A case report

**DOI:** 10.1186/1746-1596-3-36

**Published:** 2008-09-03

**Authors:** Isabel Alvarado-Cabrero, Delia Pérez-Montiel, Ondrej Hes

**Affiliations:** 1 Department of Pathology, Mexican Oncology Hospital, Instituto Mexicano del Seguro Social (IMSS), Mexico City, Mexico; 2 Department of Pathology, Instituto Nacional de Cancerologia (INCan) Av. San Fernando #22 Tlalpan, 14080 Mexico City, Mexico; 3 Department of Pathology, Laboratore Spec. Diagnostiky, Medical Faculty Hospital, Alej Svobody 80, 323 18 Pilsen, Czech Republic

## Abstract

We present the case of 80-year-old male with superficial papillary urothelial carcinoma of the urinary bladder with striking multicystic architecture with a combination of features of urothelial carcinoma with gland-like lumina, with signet-ring cell differentiation and microcystic pattern. However, the tumor shared the morphologic features of several variants of urothelial carcinoma, the most important differential diagnosis covered so-called florid Brunneriosis, cystitis cystica, and primary adenocarcinomas of the urinary bladder.

## Background

Urothelial carcinomas, particularly high-grade tumors, may show divergent differentiation. Urothelial carcinomas with true glandular differentiation must be distinguished from urothelial carcinomas with gland-like lumina. The latter represents microcystic change within otherwise typical urothelial carcinoma. Although these spaces may contain mucin, they differ from urothelial carcinoma admixed with a well-defined adenocarcinoma component [1].

Glandular neoplasms arising in the urinary bladder are an uncommon but generally recognized category of tumors. Approximately 2% of primary carcinomas in this organ account for non urachal adenocarcinomas with or without mucus production, including signet ring cell carcinomas [2,3]. We present a case of low-grade papillary urothelial carcinoma characterized by striking degrees of glandular differentiation, microcystic change and signet ring cell morphology.

## Case presentation

### Clinical Features

An 80-year-old male, a heavy smoker, was admitted to the hospital due to hematuria. Cystoscopy revealed multiple superficial small tumors ranged in size from 0.4–2 cm. Transurethral resection was performed and subsequent local chemotherapy by Mitomycin followed. The patient underwent cystoscopic resection of three recidiving superficial tumors 1 year later. The patient was alive and well without sign of disease 3 years after the second surgery.

### Pathologic Findings

**Figure 1:**
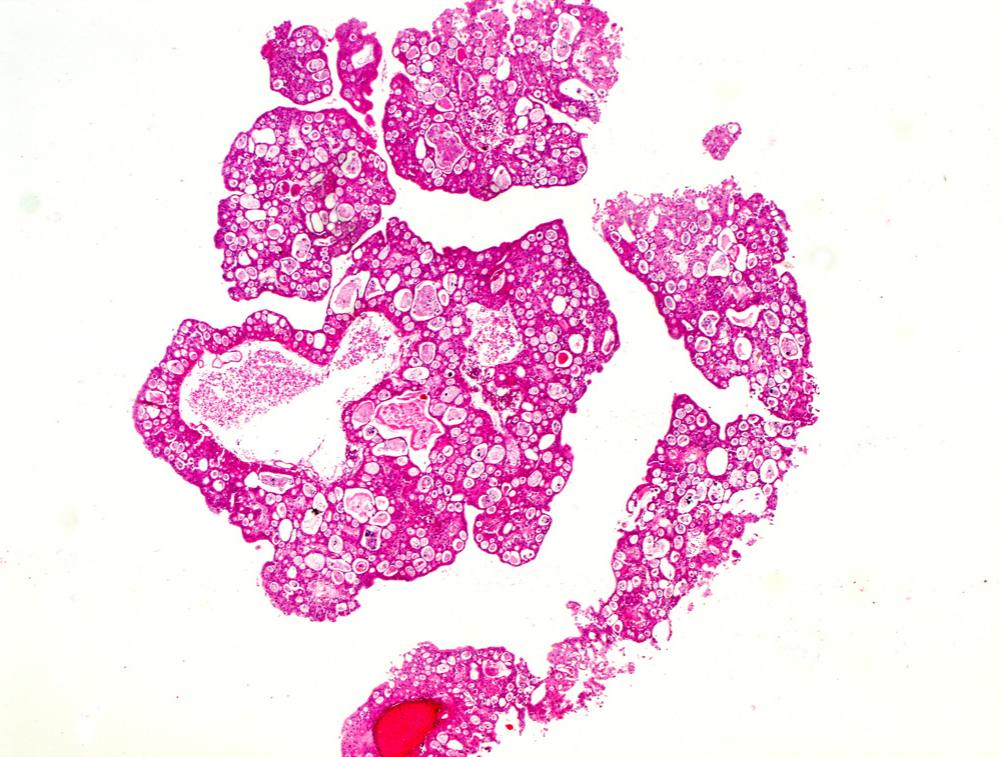
The primary tumor was characterized by multicystic and papillary architecture and the presence of gland formation (hematoxylin-eosin).

**Figure 2:**
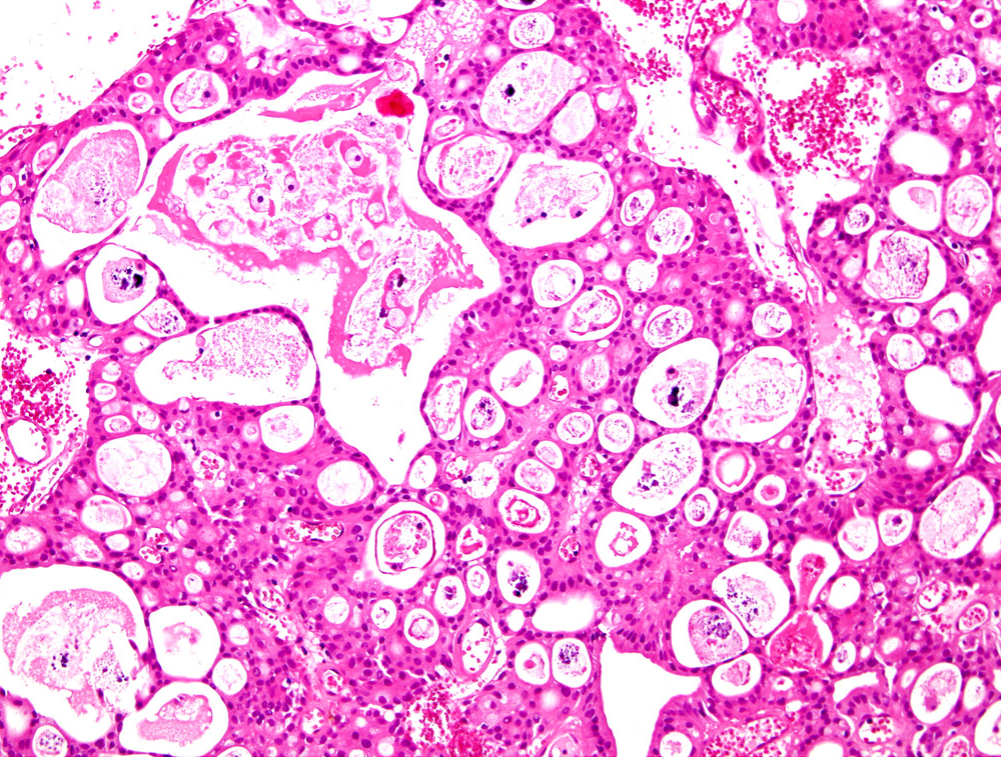
Cysts were lined with urothelial cells arranged around eosinophilic PAS (periodic acid shiff)-positive material-filled gland spaces (hematoxylin-eosin).

**Figure 3:**
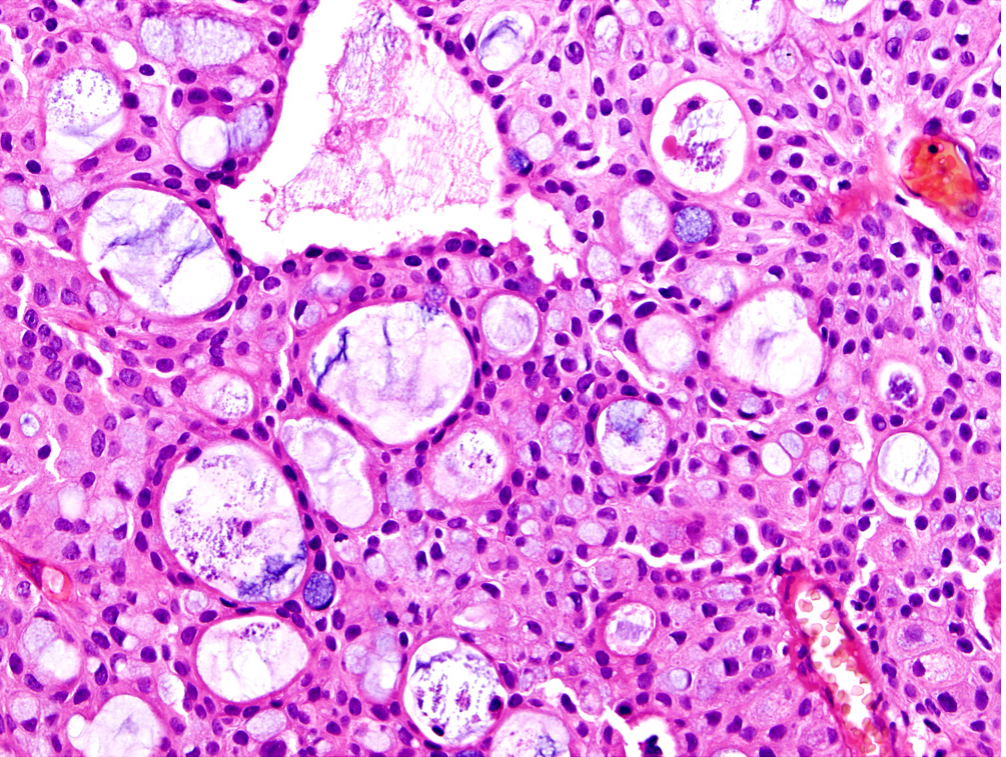
Numerous signet-ring cells were present in walls of cysts (hematoxylin-eosin).

The primary tumor was characterized by multicystic and papillary architecture and the presence of gland formation (Figure 1). Cysts were lined with urothelial cells. Some areas had columns of cells arranged concentrically around eosinophilic PAS (periodic acid shiff)-positive material-filled gland spaces (Figure 2). Additionally, numerous signet-ring cells were present in some parts (Figure 3). Areas with gland-like lumina formation were also noted. Morphology of recidiving tumor was identical to that of the primary lesion.

Tissue for light microscopy was fixed in 4% formaldehyde and embedded in paraffin using routine procedure. Sections 5-μm thick were cut from the tissue blocks and stained with hematoxylin and eosin, and periodic acid Schiff (PAS). Immunohistochemical staining was performed using the following primary monoclonal antibodies: cytokeratins AE1–AE3 (NeoMarkers, Fremont, CA, USA), CAM 5.2 (Becton Dickinson, San Jose, CA, USA), CK 7 (DakoCytomation, Glostrup, Denmark), CK 20 (DakoCytomation), LeuM1 (Becton Dickinson), cyclin D1 (DakoCytomation), carcinoembryonal antigen (DakoCytomation), cerb2 (Novocastra, Newcastle, U.K.), p53 (DakoCytomation), EGFR (DakoCytomation), MUC1 (Novocastra), MUC2 (Novocastra), and polyclonal antibody Ki 67 (DakoCytomation). Primary antibodies were visualized using the supersensitive streptavidin-biotin-peroxidase complex (Biogenex, San Ramon, CA, USA). Appropriate positive control tissues used with the primary antibodies were employed.

**Figure 4:**
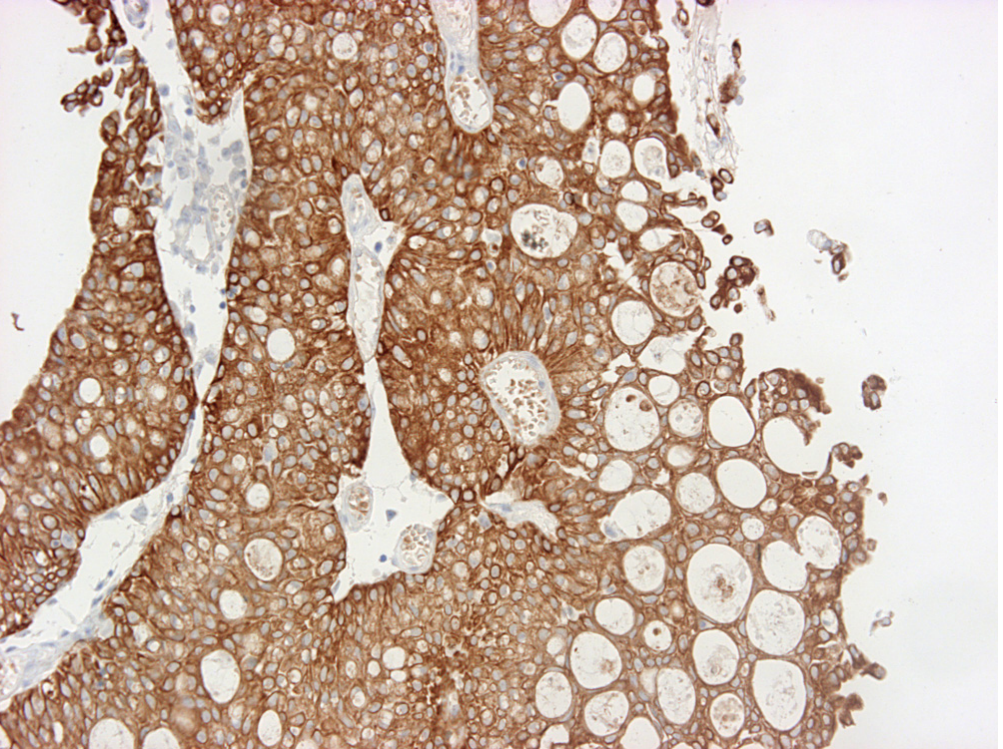
Tumor was diffusely positive for CK20 (cytokeratin 20).

**Figure 5:**
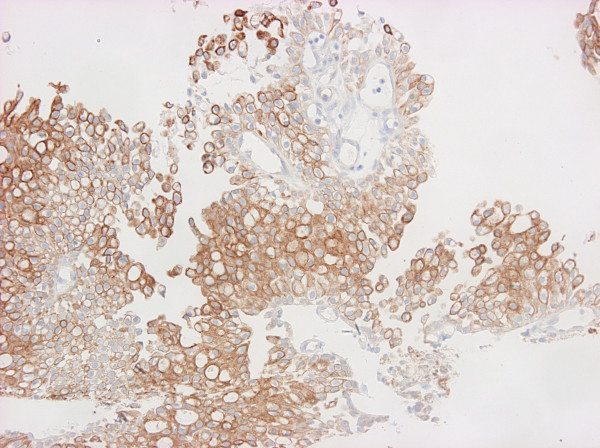
Tumor cells were also positive for CK7 (cytokeratin 7).

Moderate to strong immunoreactivity was noted for cytokeratins (CK 7, CK 20, AE1–AE3, CAM 5.2) (Figure 4 and 5), LeuM1, and cyclin, while strong but focal reactivity was revealed for CEA, cerb2 and weak focal positivity for p53. Negative reaction for epidermal growth factor receptor (EGFR) was found. The expression of MUC1 and MUC2 was strongly positive. Positive staining was restricted to either small- or intermediate-size cysts or extended to signet-ring cells, in which reactivity was predominantly cytoplasmic.

## Conclusion

Normal urothelial cells possess the capacity to produce mucoid substances [4]. Diffuse and globular mucoprotein deposits in urothelial cells have also been described [5]. The ability of urothelial cells to secrete mucin in chronic urocystitis with and without glandular metaplasia and in adenocarcinomas or signet-ring cell carcinomas of the urinary bladder is well-known [6]. On the other hand, urothelial bladder carcinoma exhibits diverse microscopic appearances. Foci of glandular metaplasia are common, usually in the form of intracytoplasmic mucin-containing vacuoles [7].

Tumor cells in our case expressed the apomucins MUC1 and MUC2 Several mucin genes designated MUC genes have been identified [8]. Previous studies have shown that MUC1 mucin protein is strongly expressed by urothelial cells. Walsh et al. examined the presence of MUC1 and -2 in normal and malignant urothelium and found that MUC1 was expressed by both normal and malignant epithelium, whereas MUC2 expression was found only in urothelial carcinomas [7]. In colon carcinomas with MUC1, gene products have been correlated with advanced disease stage [9]; on the other hand, in bladder carcinomas there is no significant association between high expression of MUC1 and histologic grade and also disease stage [7]. MUC1 and -2 expression in our case was not correlated with poor prognosis, because both primary and recidiving tumors were low-grade superficial urothelial carcinomas.

Among a total of 100 cases of urothelial carcinomas, Donhuijsen et al. found that 37 tumors revealed periodic-acid Schiff-positive cytoplasmic inclusions [5]. These inclusions were histochemically, immunohistochemically, and ultrastructurally identified as cytoplasmic deposits of mucoid materials, similar to our case.

There are several entities that should be considered in differential diagnosis. The most important reactive cystic change within the urothelium is the formation of von Brunn nests. In so-called florid Brunneriosis [10] cyst formation is more pronounced and involves a large number of nest structures. Apical glandular differentiation and eosinophilic secretion are also common, acquiring features of cystitis cystica and cystitis glandularis. The case we have described herein may show numerous cysts with mucin secretion similar to cystitis cystica.

Several overlapping morphologic variants of urothelial carcinoma with glandular appearance exist. The so-called urothelial carcinoma with tubules is composed of small- to medium-size urothelial cell-lined tubules. The growth pattern of this tumor is mainly infiltrative; moreover, it lacks the signet-ring elements and larger cystic spaces present in our case [11,12].

Another very similar entity comprises microcystic urothelial carcinoma. This type demonstrates cystic changes within an otherwise typical urothelial carcinoma, or urothelial carcinoma with glandular differentiation. Cysts were rarely be filled with eosinophilic secretion similar to our case. The cyst lining may be absent, flattened, urothelial, or differentiated toward mucinous cells. Cysts in our case were lined with urothelial cells; in addition, signet-ring cells were present in the walls of cysts [11].

Urothelial carcinoma with gland-like lumina represents another microcystic change within an otherwise typical urothelial carcinoma. These luminal structures may contain mucin [13]. We have found areas identical to this type of urothelial carcinoma within the main tumor mass of our case.

In conclusion, we present a case of superficial papillary urothelial carcinoma with striking multicystic architecture, with a combination of features of urothelial carcinoma with gland-like lumina, with signet-ring cell differentiation and microcystic pattern.

## Competing interests

The authors declare that they have no competing interests.

## Authors' contributions

IAC: has made substantial contributions to conception acquisition of data have given final approval of the version to be published. DPM: drafting the manuscript. OH: drafting and revising manuscript critically. All authors read and approved the final manuscript.

## Consent

Written informed consent was obtained from the patient for publication of this case report and any accompanying images. A copy of the written consent is available for review by the Editor-in-Chief of this journal.
